# Effects of repetitive transcranial magnetic stimulation on motor function and language ability in cerebral palsy: A systematic review and meta-analysis

**DOI:** 10.3389/fped.2023.835472

**Published:** 2023-02-16

**Authors:** Ying-Ying Sun, Lei Wang, Jin-lin Peng, Yi-jie Huang, Fu-qiang Qiao, Pu Wang

**Affiliations:** ^1^ School of Education and Psychology, University of Jinan, Jinan, China; ^2^ Jinan Tongkang Children's Hospital, Jinan, China; ^3^ Department of Rehabilitation Medicine, Hunan Provincial People's Hospital, The First Affiliated Hospital of Hunan Normal University, Changsha, China; ^4^ Tongji Hospital, Tongji Medical College, Huazhong University of Science & Technology, Wuhan, China; ^5^ Department of Rehabilitation Medicine, The Seventh Affiliated Hospital, Sun Yat-Sen University, Shenzhen, China; ^6^ CAS Key Laboratory of Mental Health, Institute of Psychology, Beijing, China

**Keywords:** cerebral palsy, motor dysfunction, rehabilitation, repetitive transcranial magnetic stimulation, non-invasive brain stimulation

## Abstract

**Objective:**

This review was conducted to assess the quality of the evidence of effectiveness of repetitive transcranial magnetic stimulation (rTMS) in treating motor and language ability of cerebral palsy (CP).

**Method:**

Medline, Cochrane library, Web of Science, Embase, PubMed, and CNKI databases were searched up to July 2021 by two independent reviewers. Randomized controlled trials (RCTs) that were published in English and Chinese and met the following criteria were included. The population comprised patients who met the diagnostic criteria for CP. Intervention included the following: comparison about rTMS and sham rTMS or comparison about rTMS combine with other physical therapy and other physical therapy. Outcomes included motor function, as follows: gross motor function measure (GMFM), Gesell Development Diagnosis Scale, fine motor function measure (FMFM), Peabody developmental motor scale, and Modified Ashworth scale. For language ability, sign-significant relation (S-S) was included. Methodological quality was assessed using the Physiotherapy Evidence Database (PEDro) scale.

**Results:**

Finally, 29 studies were included in the meta-analysis. Results of evaluation using the Cochrane Collaborative Network Bias Risk Assessment Scale showed that 19 studies specifically explained randomization, among which two studies described allocation concealment, four studies blinded participants and persons and had low risk of bias, and six studies explained that the assessment of outcome measures was blinded. Significant improvements in motor function were observed. The GMFM of total score was determined by using the random-effect model [*I*2^ ^= 88%; MD = −1.03; 95% CI (−1.35, −0.71); *P* < 0.0001] and FMFM was determined by using the fixed-effect model [*P* = 0.40 and *I*2 = 3%; SMDs = −0.48, 95% CI (−0.65, −0.30); *P* < 0.01]. For language ability, the language improvement rate was determined using a fixed-effect model [*P* = 0.88 and *I*2 = 0%; MD = 0.37, 95% CI (0.23, 0.57); *P* < 0.01]. According to the PEDro scale, 10 studies had low-quality, four studies had excellent quality, and the other studies had good quality. Using the GRADEpro GDT online tool, we included a total of 31 outcome indicators, as follows: 22 for low quality, seven for moderate quality, and two for very low quality.

**Conclusion:**

The rTMS could improve the motor function and language ability of patients with CP. However, rTMS prescriptions varied, and the studies had low sample sizes. Studies using rigorous and standard research designs about prescriptions and large samples are needed to collect sufficient evidence about the effectiveness of using rTMS to treat patients with CP.

## 1. Introduction

Cerebral palsy belongs to a group of persistent central motor and postural developmental disorders and activity limitation syndromes that are caused by non-progressive damage to the developing fetal or infant brain (before, during, or after childbirth) (1–4). CP is usually dominated by movement disorders or accompanied by disturbances in sensation, perception, cognition, communication, and behavior (4, 5). The clinical symptoms of CP caused by various etiology before birth up to the neonatal period mostly occurred before 18 months after birth; symptoms of CP caused by brain injury (hypoxia, trauma, poisoning, central nervous system infection, and others) after neonatal period and infancy period are related to the time of brain injury (4). Clinically, these are generally divided into spasticity, dyskinetic, and ataxia according to the mode of movement disorder (1, 6, 7).

Pathological changes in the brain affected by CP are characterized by abnormal brain development, brain damage caused by brain hypoxia, and intracranial hemorrhage (8, 9). The characterization of CP by delayed gross motor responses and difficulty executing movements due to dystonia, muscle weakness, and insufficient muscle coordination (10). Spasms and abnormal motor postures increase energy expenditure and hinder the normal muscle growth during development, leading to secondary muscle and soft tissue contracture and skeletal deformities (11). Children with CP with these movement disorders have functional impairments in activities of daily living (ADL) and ability of self-care (e.g., dressing and feeding) and mobility (12). In Europe, the prevalence of CP was 1.5–3/1,000 births (13). Achieving independence in self-care and mobility is the goal for children with CP.

The most common cause of CP is white matter damage in the brain. CP is a non-progressive disease that leads to worsening of clinical features with the abnormal development of the central nervous system if left unchecked (14). To obtain an effective and long-lasting therapeutic effect, the therapeutic measures need to have a function that affects the neuroplasticity of the brain in the long term (15). Currently, research in the field of pediatric neurology has focused on the efficacy of non-invasive brain stimulation (NIBS) in the treatment of various pediatric neurological disorders (16). NIBS is a means of inducing electrical currents in brain tissue with the effect of promoting immediate and long-term modulation of motor cortex excitability (17). Therefore, it is a non-drug management candidate strategy for the treatment of pediatric movement disorders (18, 19).

As a technique kind of NIBS, the technical features of rTMS are non-invasive and painless (20, 21), which applies electromagnetic principles to brain regions (22), and adjusts the function of various areas of the cerebral cortex by changing the excitability of neurons. rTMS has achieved remarkable therapeutic effects in the treatment of neurological diseases, such as stroke and autism spectrum disorder (23) and has gradually become an important technique for the treatment of these diseases (24).

Nowadays, rTMS is used in the treatment of children with CP increasingly (25). rTMS can improve motor function (26), relieve spasm (27),restore the speech function of patients with CP (28) and can change brain function by modulating developmental plasticity (29). However, studies on rTMS varied in sample size and thus show different results. High-quality evidence-based medical studies that systematically evaluated the efficacy of rTMS in the treatment of CP remain few.

Thus, summarizing studies based on rTMS-related factors is critical to the accurate estimation of the effects of rTMS on CP. The aims of this meta-analysis were as follows: to systematically evaluate the quality and efficacy of rTMS in alleviating motor dysfunction and restoring speech ability in patients with CP according to randomized clinical trials (RCTs); and to search for strong evidence for the effectiveness of using rTMS for CP.

## 2. Methods

This systematic review was planned and conducted according to the Preferred Reporting Items for Systematic Reviews and Meta-Analyses Guideline and Cochrane Collaboration (30).

### 2.1. Search strategy

Two reviewers (Ying-Ying Sun and Lei Wang) performed electronic searches in the following publication databases in July 2021 without restrictions on publication year: Medline, Cochrane Library, Web of Science, Embase, PubMed, and China National Knowledge infrastructure (CNKI). Various combinations of keywords or subject words were used as search terms, including the following: “TMS,” “transcranial magnetic stimulation,” “non-invasive brain stimulation,” “cerebral palsy,” and “CP.” Pre-searches were performed. Then, the final search style was selected as follows: PUBMED: “((Cerebral Palsy[Title]) OR (Cerebral Palsy[MeSH Terms])) AND ((((((repetitive transcranial magnetic stimulation[MeSH Terms]) OR (repetitive transcranial magnetic stimulation[Title])) OR (rTMS[Title])) OR (rTMS[MeSH Terms])) OR (repetitive TMS[MeSH Terms])) OR (repetitive TMS[Title])).” The number of manual searches were increased to complement the results and to reduce the number of articles that may have been missed by electronic database searches.

### 2.2. Eligibility criteria

The Population, Intervention, Comparison, Outcomes, Study Design (PICOS) framework was used to determine the eligibility criteria of the articles to be included in the review. The population included patients who met the diagnostic criteria for CP. Participants clearly stated in the included literature that the compliance or diagnosis was CP and that they were aged under 18 years old. For intervention, the studies using rTMS as intervention and with a well-defined protocol that involved information on the specific training parameters (type, time, intensity, frequency, and duration) were included. For comparison, the experimental groups received rTMS (low- or high-frequency rTMS) or rTMS combined with other physical therapies. The control group received sham TMS or other types of physical therapy. The outcomes (for meta-analysis) were measured by using gross motor function measure (GMFM), Gesell Development Diagnosis Scale (GDDS), fine motor function measure (FMFM), Peabody developmental motor scale (PDMS), and Modified Ashworth Scale (MAS). For language ability, sign-significant relation (S-S) was included. For the study design, only RCTs were included in the review.

### 2.3. Exclusion criteria

Studies involving animal research, conference paper, protocol study or computer model research, and duplicate papers were excluded. Two authors (Ying-Ying Sun and Yi-jie Huang) independently reviewed the title and abstract sections of the retrieved articles. First, we eliminated duplicate articles by using “Medical Literature King V6” software. Second, we excluded inappropriate articles after reading the title and abstract following the eligibility criteria in the PICOS framework (16). Finally, we downloaded potentially relevant articles for a more detailed full-text review. If the results of the two independent authors differed, then the third author (Pu Wang) participated in the discussion, and final consensus was reached.

### 2.4. Data extraction

We extracted the following data: general information including first author, year of publication, sample size, gender, age, treatment course, and intervention measures; outcome indicators including GMFM, GDDS, FMFM, PDMS, and MAS; and language ability, S-S. The collection of data and general information were conducted by two authors (Ying-Ying Sun and Yi-jie Huang).

### 2.5. Quality assessment

The methodological quality of the intervention studies was assessed using the Physiotherapy Evidence Database (PEDro) scale (25). According to the PEDro scale, the quality of papers were classified: studies with scores of lower than six points were considered low-quality studies (scores <6), good-quality studies (scores = 6 or 7), and excellent -quality studies(scores >7) (31).

GRADEpro GDT online tool was used in evaluating the level of evidence quality of outcome indicators. The indicators of outcome quality included five degrading factors, namely, risk of bias, inconsistency, indirectness, imprecision, and other considerations. The quality of evidence can be divided into four levels, namely, “high,” “moderate,” “low,” and “very low.”

Two reviewers (Ying-Ying Sun and Jin-lin Peng) independently evaluated the quality of the included studies, If the results of the two independent authors differ, then a third author (Pu Wang) participated in the discussion and decided the final consensus.

### 2.6. Risk of bias assessment in individual studies

To minimize errors and potential biases in the evaluation, the quality of the included studies was evaluated, and their scores were compared in a consensus meeting between two independent authors (Wang Lei and Fu-qiang Qiao). In case of disagreements, a third author (Pu Wang) was included in the discussion to achieve a final consensus. The Cochrane risk of bias assessment tool was used to assess the risk of bias of these articles. Each article was assessed for selection, performance, detection, attrition, and reporting biases. Each domain was rated as having high risk of bias, unclear bias, or low risk of bias. The risk map of bias of these studies' quality was prepared with RevMan 5.3 software.

### 2.7. Statistical analysis

The Review Manager 5.2 software of Cochrane Collaboration was used in the meta-analysis. The outcome variables were continuous. Thus, the mean difference (MD) was calculated, and the 95% CI of the statistical results was reported. A *P* value of less than 0.05 indicated statistical significance for an overall effect (Z). Chi-square test was used to calculate the heterogeneity of the included articles. When heterogeneity was *P* > 0.1 and *I*2^ ^< 50%, a fixed-effect model was used. When heterogeneity was *I*2^ ^> 50%, the causes of heterogeneity were analyzed by subgroup or sensitivity analysis. When the results still had heterogeneity, the random-effect mode was used for summary analysis.

## 3. Results

### 3.1. Search results

**Figure 1:**
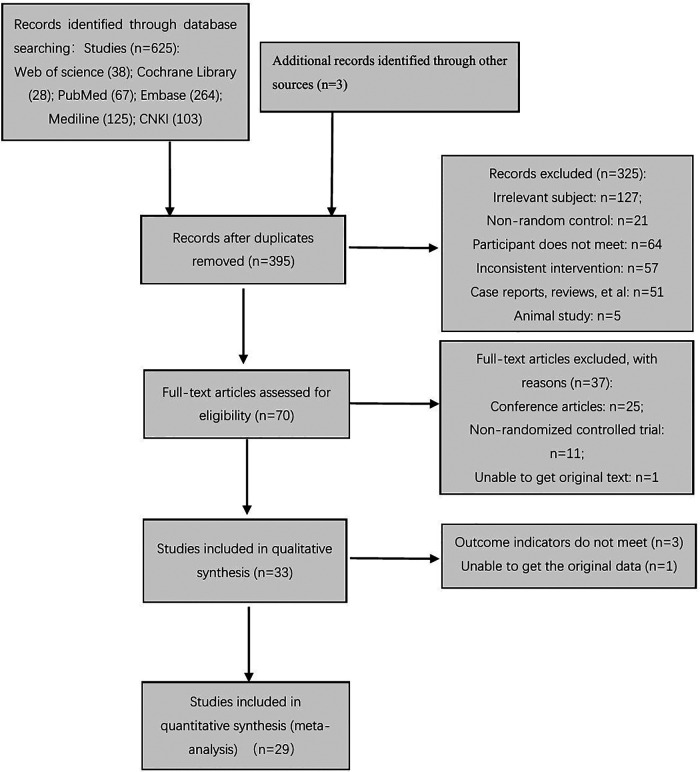
Flow chart of the search process.

At different stages of retrieval and screening, different numbers of studies were excluded. The detailed reasons and procedures are shown in Figure 1. A total of 625 abstracts were retrieved, and all were imported into the Document Management Software of “Medical Literature King V6.” A total of 230 duplicate studies were eliminated, and 325 studies were excluded after reading the titles and abstracts. Seventy studies were left after the screening process, which involved reading the abstracts. The full texts were downloaded for further screening. Twenty-five studies were excluded, because they were conference articles. Eleven studies were excluded, because they included non-randomized controlled trials. One study was excluded, because it did not contain original text. After excluding the abovementioned studies, 33 studies were included in the qualitative analysis. After the article outcome indicators were read, three studies (26, 32, 33) were excluded, because their outcome indicators did not meet the inclusion criteria. The data of one study (34) only reported the *P*-value, and the original data were not obtained even after contacting the studies' authors; thus, the requirement for data analysis of the meta-analysis was not met. Finally, 29 studies were included in the meta-analysis.

### 3.2. Assessment of quality

The studies evaluated according to the PEDro scale are listed in Table 1. Ten studies had low quality (27, 35, 41, 43, 44, 48, 50, 51, 55, 56), four studies had excellent quality (26, 32–34), and the other studies had good quality.

**Table 1:** The studies evaluated according to the PEDro scale.

Pedro Scale Questions	Q1	Q2	Q3	Q4	Q5	Q6	Q7	Q8	Q9	Q10	Q11	Total Score
Wang et al (35)	Y	N	N	Y	N	N	N	Y	Y	Y	Y	5
Liang et al (36),	Y	Y	N	Y	N	N	Y	Y	Y	Y	Y	7
Li et al (37),	Y	Y	N	Y	N	N	N	Y	Y	Y	Y	6
Wang and Zhou (38),	Y	Y	N	Y	N	N	Y	Y	Y	Y	Y	7
Zhang et al (39)	Y	Y	N	Y	N	N	N	Y	Y	Y	Y	6
Wu et al (40),	Y	Y	N	Y	N	N	N	Y	Y	Y	Y	6
Zhang et al (1) (41),	Y	N	N	Y	N	N	N	Y	Y	Y	Y	5
Li et al (1) (42)	Y	Y	N	Y	N	N	N	Y	Y	Y	Y	6
Liang (43),	Y	N	N	Y	N	N	N	Y	Y	Y	Y	5
Xu (44),	Y	N	N	Y	N	N	N	Y	Y	Y	Y	5
Wang (45),	Y	N	N	Y	N	N	Y	Y	Y	Y	Y	6
Li et al (46),	Y	Y	N	Y	N	N	N	Y	Y	Y	Y	6
Deng et al (47),	Y	Y	N	Y	N	N	N	Y	Y	Y	Y	6
Li (48),	Y	N	N	Y	N	N	N	Y	Y	Y	Y	5
Bai et al (49),	Y	Y	N	Y	N	N	N	Y	Y	Y	Y	6
Ma and Ye (50),	Y	N	N	N	N	N	N	Y	Y	Y	Y	4
Zhang and Ding (51),	Y	N	N	Y	N	N	N	Y	Y	Y	Y	5
Bao and Liu (52),	Y	Y	N	Y	N	N	N	Y	Y	Y	Y	6
Qiu (53)	Y	Y	N	Y	N	N	N	Y	Y	Y	Y	6
Duan (54)	Y	Y	Y	Y	N	N	N	Y	Y	Y	Y	7
Cao (55),	Y	N	N	Y	N	N	N	Y	Y	Y	Y	5
Feng et al (56),	Y	N	N	Y	N	N	N	Y	Y	Y	Y	5
Fan e al (57),	Y	Y	N	Y	N	N	N	Y	Y	Y	Y	6
Xu et al (58)	Y	Y	N	Y	N	N	N	Y	Y	Y	Y	6
Wang and Ma (59),	Y	Y	N	Y	N	N	N	Y	Y	Y	Y	6
Qiu et al (60),	Y	Y	N	Y	N	N	N	Y	Y	Y	Y	6
Zhang (61),	Y	Y	N	Y	N	N	N	Y	Y	Y	Y	6
Yang et al (62),	Y	Y	N	Y	N	N	N	Y	Y	Y	Y	6
Gillick et al (26),	Y	Y	N	Y	Y	Y	Y	Y	Y	Y	Y	9
Valle et al (32)	Y	Y	n	Y	Y	Y	Y	y	y	y	y	9
Kirton et al (33),	Y	Y	N	Y	Y	Y	N	Y	Y	Y	Y	8
Gupta et al (27)	Y	N	N	Y	N	N	N	Y	Y	Y	Y	5
Kirtonet al (34)	Y	Y	Y	Y	Y	N	N	Y	Y	Y	Y	8

### 3.3. Risk of bias assessment in individual studies

**Figure 2:**
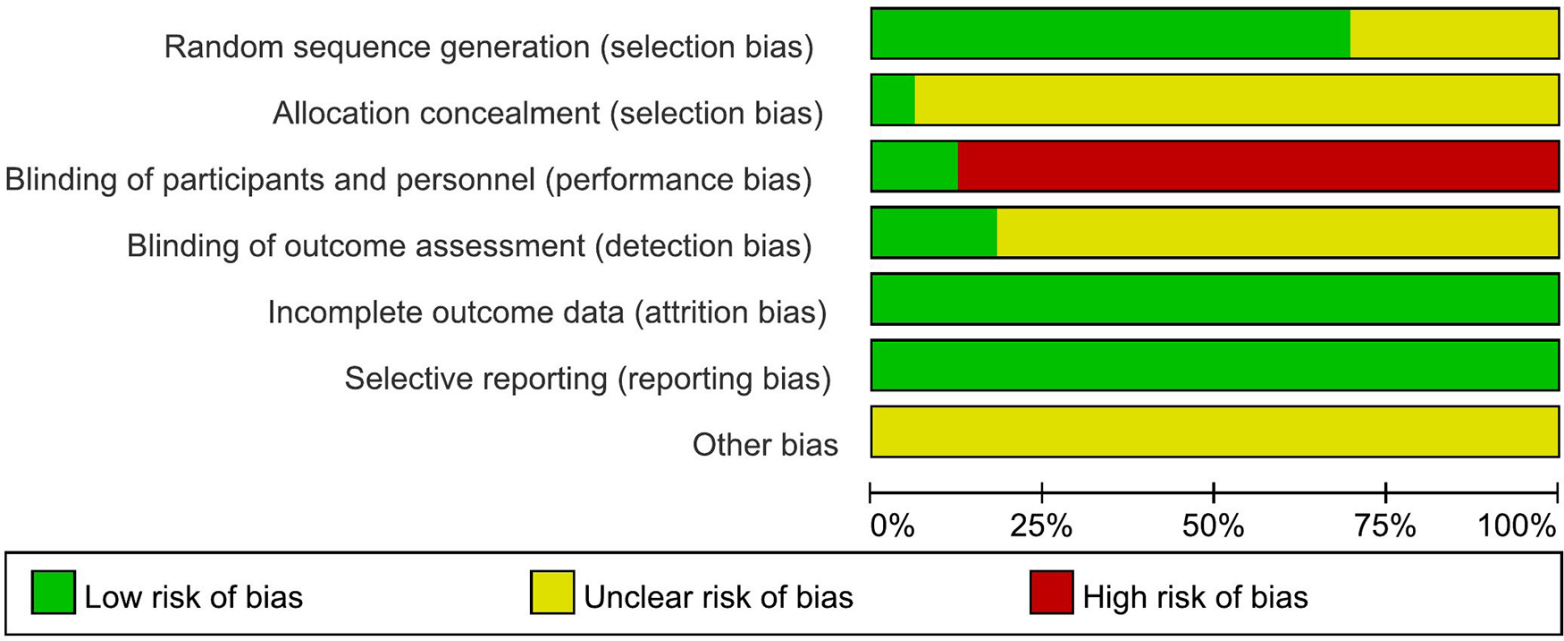
Risk of bias graph.

**Figure 3:**
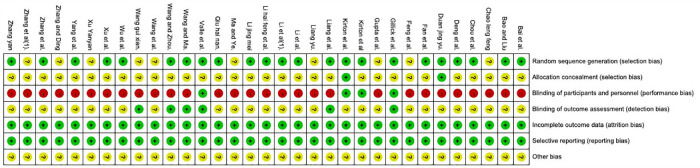
Risk of bias graph.

The results of risk of bias are shown in Figures 2, 3. Nineteen studies specifically explained the random methods used, 10 studies (27, 35, 41, 43–45, 50, 51, 55, 56) did not report random sequence generation, two studies (34, 54) described allocation concealment. Four studies (26, 32–34) blinded the participants and persons and had low risk of bias, because the intervention method was rTMS vs. sham rTMS. Six studies (26, 32, 36, 38, 45, 59) explained that the assessment of outcome measures was blinded. Reporting and attrition biases were low risk of bias.

### 3.4. Study characteristics

As shown in Table 2, the characteristics included in the studies were first author, sample size, age, gender, and diagnosis criteria. As shown in Table 3, the characteristics included in the studies were content of intervention program, site of stimulation, duration of rTMS, number of rTMS sessions, outcomes measured, and assessment time points.

**Table 2:** Study characteristics.

Number	Patients	Age (C/T, year)	sample (w/m)	Treatment Group *n* (F/M)	Control Group *n* (F/M)	Diagnosis criteria
Wang et al (35)	Cerebral palsy	2.9 ± 0.2 3.1 ± 0.2	28/14	13/8	15/6	Rehabilitation Guidelines for Cerebral Palsy in China (2015)
Liang et al (36),	Cerebral palsy	27.35 ± 9.01 (m); 25.37 ± 10.28 (m)	33/27	17/13	16/14	Rehabilitation Guidelines for Cerebral Palsy in China (2015)
Li et al (37),	Cerebral palsy		75	38	37	Rehabilitation Guidelines for Cerebral Palsy in China (2006)
Wang and Zhou (38),	Cerebral palsy complicated with epilepsy	22.74 ± 8.51 (m) 22.43 ± 9.48 (m)	146/124	71/59	75/65	Rehabilitation Guidelines for Cerebral Palsy in China (2006)
Zhang et al (39)	Spastic hemiplegic cerebral palsy	4.6 ± 1.6 4.5 ± 1.8	28/8	15/5	13/3	Not specified
Wu et al (40),	Spastic cerebral palsy	2.9 ± 0.9 3.2 ± 0.6	30/24	16/11	14/13	Not specified
Zhang et al (1) (41),	Spastic cerebral palsy	7 ± 1.59 6.98 ± 1.56	29/11	16/4	13/7	Not specified
Li et al (1) (42)	Spastic hemiplegic cerebral palsy	24.9 ± 5.5 (m) 25.5 ± 4.4 (m)	25/5	12/3	13/2	Not specified
Liang (43),	Spastic hemiplegic cerebral palsy	4.5 ± 0.8 4.4 ± 1.0	43/37	21/19	22/18	Discussion on guidelines for diagnosis and treatment of cerebral palsy in children
Xu (44),	Cerebral palsy	45.01 ± 8.25 (m) 43.87 ± 7.25 (m)	44/54	22/28	22/26	Children with cerebral palsy
Wang (45),	Cerebral palsy complicated with epilepsy	24.68 ± 8.60 (m) 24.59 ± 8.57 (m)	41/40	21/22	20/18	Chinese Cerebral Palsy Rehabilitation Guidelines
Li et al (46),	Cerebral palsy	19.4 ± 8.7 (m) 20.7 ± 7.4 (m)	36/19	19/9	17/10	Relevant diagnostic criteria adopted by the Second National Children's Rehabilitation and the Ninth National Pediatric Cerebral Palsy Academic Conference in 2006
Deng et al (47),	Cerebral palsy	4.4 ± 2.2 4.6 ± 2.4	38/26	20/12日	18/14	Not specified
Li (48),	Cerebral palsy	5.43 ± 1.63 5.28 ± 1.46	45/41	22/21	23/20	The definition, classification and diagnostic conditions of cerebral palsy in children
Bai et al (49),	Spastic cerebral palsy	4.0 ± 0.4 4.0 ± 0.5	156/144	79/71	77/73	Rehabilitation Guidelines for Cerebral Palsy in China (2015)
Ma and Ye (50),	Cerebral palsy	5.0 ± 1.7	34/26	30	30	Rehabilitation Guidelines for Cerebral Palsy in China (2014)
Zhang and Ding (51),	Cerebral palsy	6–72 (range) 5–60 (range)	52/38	27/13	25/15	Rehabilitation Guidelines for Cerebral Palsy in China (2006)
Bao and Liu (52),	Cerebral palsy	36.64 ± 18.14 (m) 36.03 ± 13.11 (m)	25/20	12/10	13/10	The diagnosis and classification criteria of USCP developed by the Pediatric Neurology Group of the Pediatrics Branch of the Chinese Medical Association
Qiu (53)	Cerebral palsy	31 ± 9 (m) 28 ± 7 (m)	60/36	31/17	29/19	The diagnostic criteria for cerebral palsy formulated by the Second National Children's Rehabilitation and the Ninth National Pediatric Cerebral Palsy Rehabilitation Academic Conference in 2006
Duan (54)	Cerebral palsy	32. 1 ± 12. 6 (m) 32. 5 ± 16. 1 (m)	131/79	64/41	67/38	The 13th National Pediatric Cerebral Palsy Rehabilitation Academic Conference and International Academic Exchange Conference Diagnostic Criteria for Children with Cerebral Palsy
Cao (55),	Cerebral palsy	33.70 ± 16.79 (m) 33.07 ± 17.35 (m)	57/45	29/22	28/23	The diagnostic criteria proposed by the Child Rehabilitation Professional Committee of the Chinese Society of Rehabilitation Medicine and the Chinese Disabled Rehabilitation Association Pediatric Cerebral Palsy Rehabilitation Professional Committee (2006)
Feng et al (56),	Spastic cerebral palsy	30 ± 10 (m) 28 ± 7 (m)	58/17	33/9	25/8	The diagnostic criteria proposed by the Child Rehabilitation Professional Committee of the Chinese Society of Rehabilitation Medicine and the Chinese Disabled Rehabilitation Association Pediatric Cerebral Palsy Rehabilitation Professional Committee (2006)
Fan e al (57),	Cerebral palsy	2.7 ± 0.8 2.5 ± 0.7	44/37	22/19	22/18	Not specified
Xu et al (58)	Cerebral palsy	4.92 ± 0.33 4.55 ± 0.16	32/18	17/8	15/10	Diagnostic criteria adopted by the Ninth National Pediatric Cerebral Palsy Rehabilitation Academic Conference (2006)
Wang and Ma (59),	Cerebral palsy complicated with epilepsy	20.57 ± 6.54 (m) 19.28 ± 7.81 (m)	88/68	49/37	39/31	The diagnostic criteria proposed by the Child Rehabilitation Professional Committee of the Chinese Society of Rehabilitation Medicine and the Chinese Disabled Rehabilitation Association Pediatric Cerebral Palsy Rehabilitation Professional Committee (2006)
Qiu et al (60),	Hypotonic Cerebral Palsy	15.1 ± 5.3 (m) 14.5 ± 4.0 (m)	38/19	17/10	21/9	Not specified
Zhang (61),	Cerebral palsy with language development disorder	4.1 ± 1.3 4.2 ± 1.2	57/63	29/31	28/32	Not specified
Yang et al (62),	Cerebral palsy with language development disorder	4.6–1.5 4.8 ± 1.7	49/15	24/8	25/7	Standards for the National Symposium on Children with Cerebral Palsy (2004)
Gillick et al (26),	Pediatric hemiparesis	10 years 10 mmonths ± 2 yyears 10 months	10/9	5/5	5/4	Not specified
Valle et al (32)	Cerebral palsy	9 years 1month ± 3 years 2 months	9/8	7/4	4/2	(1980) Cerebral palsy diagnosis in children over age1 year: standard criteria
Kirton et al (33),	Cerebral palsy	median age 13·25	6/4	3/2	3/2	Not specified
Gupta et al (27)	cerebral palsy	8.11 ± 4.09 7.93 ± 4.85	20	10	10	Not specified
Kirtonet al (34)	cerebral palsy	10.34 ± 3.5 12.2 ± 4.2	10/10	5/5	5/5	Not specified

**Table 3:** Study characteristics.

Number	Content of Intervention Program	Site of Stimulation	Duration of TMS	Number of TMS Sessions	Outcomes Measured	Assessment Time Points
Wang et al (35)	C: Routine rehabilitation T:C + rTMS	The cerebral hemisphere motor cortex	5HZ, 20 min	once per day, 5 days of treatment per week, continuous treatment for 8 weeks	GMFM, GDDS	After 2 months of treatment
Liang et al (36),	C: Routine rehabilitation + CIMT T:C + rTMS	the contralateral cerebral motor cortex	1HZ, 20 min	once per day, 5 days of treatment per week, continuous treatment for 4 weeks	PDMS; UEFT	After 4 weeks of treatment
Li et al (37),	C: Routine rehabilitation T:C + rTMS	Bilateral cerebral motor cortex	5HZ, 20 min	Once per day, Continuous treatment for 2 weeks is a course of treatment, with a 10d interval between the 2 courses of treatment, and a total of 4 courses of treatment.	GMFM; FMEM	After 3 months of treatment
Wang and Zhou (38),	C: Routine rehabilitation T:C + rTMS	prescription of epilepsy treatment	0.5HZ, 20 min	Once per day, continuous treatment for 2 months.	GMFM, PDMS	After 2 months of treatment
Zhang et al (39)	C: Routine rehabilitation T:C + rTMS	Contralateral motor cortex	5HZ, 20 min	Once per day , 15 days/course of treatment, rest for 5 days after each course of treatment, 3 consecutive courses of treatment.	MAS; FMFM	After 3 months of treatment
Wu et al (40),	C: Routine rehabilitation T:C + rTMS	Bilateral cerebral motor cortex	5HZ, 20 min	Once per day, continuous treatment for 1 month.	GMFM; S-S	After 4 weeks of treatment
Zhang et al (1) (41),	C: Routine rehabilitation T:C + rTMS	Bilateral cerebral motor cortex	5HZ, 20 min	once per day, 5 days of treatment per week, continuous treatment for 4 weeks.	GMFM	After 5 weeks of treatment
Li et al (1) (42)	C: Routine rehabilitation T:C + rTMS	Contralateral motor cortex	1HZ, 20 min	once per day, 5 days of treatment per week, continuous treatment for 4 weeks.	PDMS ; FMFM	After 4 weeks of treatment
Liang (43),	C: Routine rehabilitation T:C + rTMS	Bilateral cerebral motor cortex	5HZ, 20 min	once per day, 5 days of treatment per week, continuous treatment for 3 months.	GMFM; S-S	After 3 months of treatment
Xu (44),	C: Routine rehabilitation + Virtual reality training T:C + rTMS	Contralateral motor cortex	1HZ, 20 min	once per day, 5 days of treatment per week, continuous treatment for 4 weeks.	PDMS; GMFM; MAS	After 4 weeks of treatment
Wang (45),	C: Routine rehabilitation T:C + rTMS	prescription of epilepsy treatment	0.5HZ, 20 min	Once per day, continuous treatment for 2 months.	GMFM	After 2 months of treatment
Li et al (46),	C: Routine rehabilitation T:C + rTMS	Bilateral cerebral motor cortex	5HZ, 20min	Once per day, 5 days of treatment per course of treatment, with a 10d interval between the next courses of treatment, continuous treatment for 3 months.	GDDS; PDMS	After 3 months of treatment
Deng et al (47),	C: Routine rehabilitation T:C + rTMS	Bilateral cerebral motor cortex	5HZ, 20 min	once per day, 5 days of treatment per week, continuous treatment for 3 months.	GMFM; PDMS	After 3 months of treatment
Li (48),	C: Routine rehabilitation T:C + rTMS	Bilateral cerebral motor cortex	5HZ, 20 min	Once per day, continuous treatment for 180 days.	GMFM; PDMS	After 180 days of treatment
Bai et al (49),	C: Routine rehabilitation T:C + ILF-TMS	Not specified	0.2HZ,30 min	Once per day, 5 days of treatment per course of treatment, with a 3 d interval between the next courses of treatment, continuous treatment for 3 months.	WAS; GMFM	After 3 months of treatment
Ma and Ye (50),	C: Routine rehabilitation T:C + rTMS	Bilateral cerebral motor cortex	10HZ, 20 min	once per day, 5 days of treatment per week, continuous treatment for 3 months.	GMFM	After 3 months of treatment
Zhang and Ding (51),	C: Routine rehabilitation T:C + rTMS	Not specified	6 frequencies conversion (5, 10, 20, 30, 40, 50 Hz) 30min	Once per day, 20 days of treatment per course of treatment, with a 10 d interval between the next courses of treatment, continuous treatment for 3 months.	GMFM	After 3 months of treatment
Bao and Liu (52),	C: Routine rehabilitation T:C + rTMS	Bilateral frontal lobes	1HZ, 600 plus	once per day, 5 days of treatment per week, continuous treatment for 1 month.	MAS; UEFT	After one months of treatment
Qiu (53)	C: Routine rehabilitation + Acupuncture T:C + ILF-TMS	Not specified	0.2HZ, 30 min	once per day, 5 days of treatment per week, continuous treatment for 1 month.	GMFM	After one months of treatment
Duan (54)	C: Routine rehabilitation T:C + ILF-TMS	Not specified	50GS, 30 min	One per day, continuous treatment for 3 months.	GMFM; FMFM; S-S	After 3 months of treatment
Cao (55),	C: Routine rehabilitation T:C + ILF-TMS	Not specified	50GS, 30 min	One per day, continuous treatment for 30 days.	GMFM;FMFM, S-S	After 30 days of treatment
Feng et al (56),	C: Routine rehabilitation T:C + ILF-TMS	Not specified	<450GS, 30 min	One per day, continuous treatment for 3 months.	GMFM; FMFM	After 3 months of treatment
Fan e al (57),	C: Routine rehabilitation T:C + rTMS	Bilateral frontal, temporal, and occipital lobes	5HZ, 20 min	Two per day, 5 days of treatment per week, continuous treatment for 3 months.	GDDS	After 3 months of treatment
Xu et al (58)	C: Routine rehabilitation T:C + rTMS	Not specified	20 min	Two per day, 5 days of treatment per week, continuous treatment for 6 months.	S-S	After 6 months of treatment
Wang and Ma (59),	C: Routine rehabilitation T:C + rTMS	prescription of epilepsy treatment	5HZ, 30 min	Once per day, continuous treatment for 4 months.	GDDS	After 4 months of treatment
Qiu et al (60),	C: Routine rehabilitation T:C + rTMS	4th and 5th lumbar intervertebral space	30HZ, 20 min	Once per day, two weeks of treatment per course of treatment, with two weeks interval between the next courses of treatment, continuous treatment for 2 months.	GDDS	After 2 months of treatment
Zhang (61),	C: Routine rehabilitation T:C + rTMS	Not specified	20 min	Two per day, 5 days of treatment per week, continuous treatment for 3 months.	S-S; PDMS	After 3 months of treatment
Yang et al (62),	C: Routine rehabilitation T:C + rTMS	Not specified	20 min	Once per day, 5 days of treatment per week, continuous treatment for 3 months.	S-S	After 3 months of treatment
Gillick et al (26),	C: CIMT + sham rTMS T: CIMT + Real rTMS	The ipsilesional primary motor cortex	1HZ, 600 plus	Five treatments of rTMS for 2 weeks.	AHA, COPM	After 2 weeks of treatment
Valle et al (32)	C: CIMT + sham rTMS T: CIMT + Real rTMS (1HZ) T: CIMT + Real rTMS (5HZ)	the motor cortex	1HZ, 5HZ, 1500 plus	Five consecutive daily sessions	MAS; ROM	After 5 days of treatment
Kirton et al (33),	C: sham rTMS T: Real rTMS	over contralesional motor cortex	1HZ, 20 min	Once per day for 8 days	UEFT	After 5 days of treatment
Gupta et al (27)	C: Physical therapy T:C + rTMS	Not specified	5 Hz frequency for 15 min	Once per day for 20 days (5 days a week for 4 weeks)	GMFM.	After 4 weeks of treatment
Kirtonet al (34)	C: sham rTMS T: Real rTMS	The contralesional primary motor cortex	1 Hz, and duration 20 min	Once per day for 20 days (5 days a week for 2 weeks)	AHA, COPM	After 2 weeks of treatment

### 3.5. Outcomes

#### 3.5.1. GMFM

The GMFM consists of 88 items grouped into five domains, namely, (A) lying and rolling, (B) sitting, (C) crawling/kneeling, (D) standing, and (E) walking/running/jumping. The analysis was performed according to the five domains and total score.

##### 3.5.1.1. A

**Figure 4:**
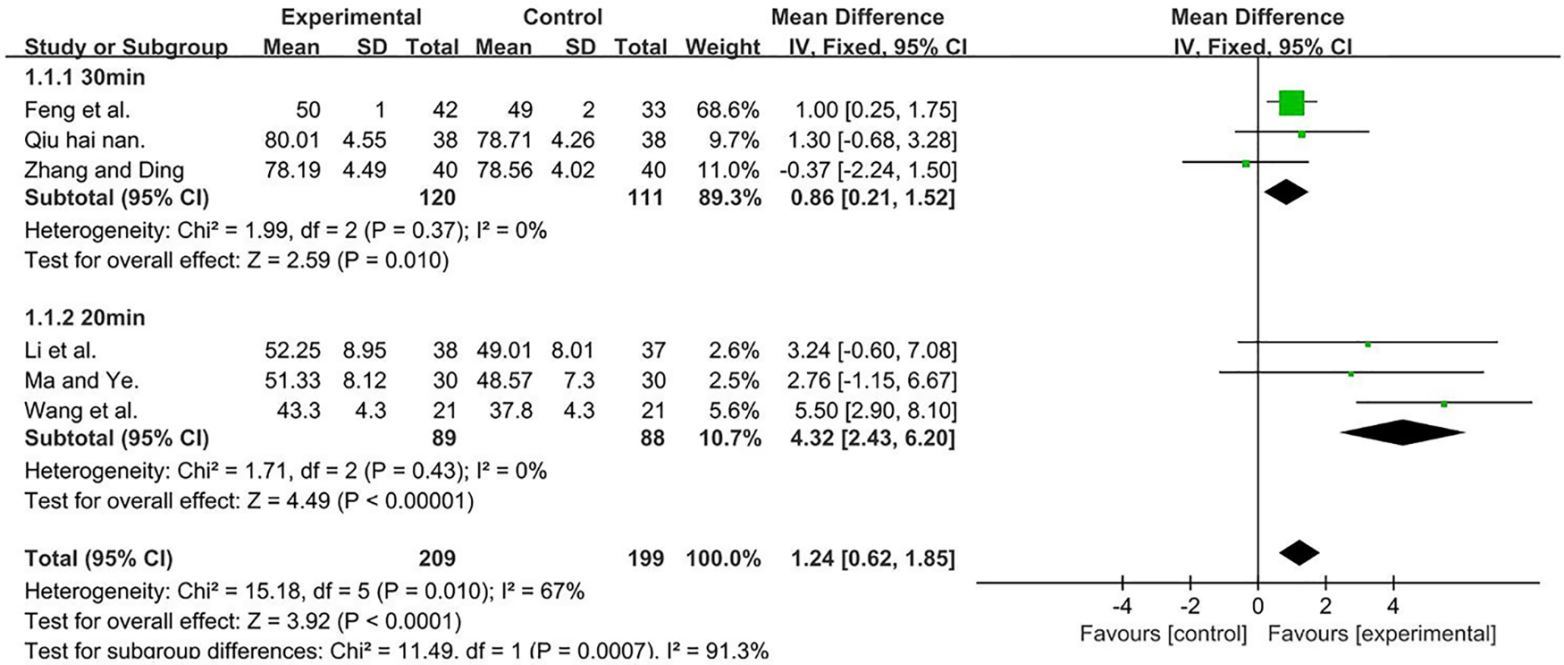
Forest plot showing MD (with 95% CI) for GMFM-A of the included studies comparing the experimental and control groups. Note: For the 30 min subgroup, *P* = 0.37 and *I*^2^ = 0%, fixed-effect model [MD = 0.86, 95% CI (0.21, 1.52); *P* = 0.01]. For the 20 min subgroup, *P* = 0.43 and *I*^2^ = 0%, fixed-effect model [MD = 4.32; 95% CI (2.43, 6.20); *P* < 0.001]. The analysis results of both subgroups were statistically significant.

A total of 408 participants were included in six studies (35, 37, 50, 51, 53, 56) [*I*2_ _= 67%; MD = 1.86, 95% CI (0.38, 3.35); *P* = 0.01]. We performed a subgroup analysis, because heterogeneity was observed. According to the duration of TMS, the group was divided into two subgroups, namely, 30 min (51, 53, 56) and 20 min (35, 37, 50), and the result favors rTMS, as shown in Figure 4.

##### 3.5.1.2. B

**Figure 5:**
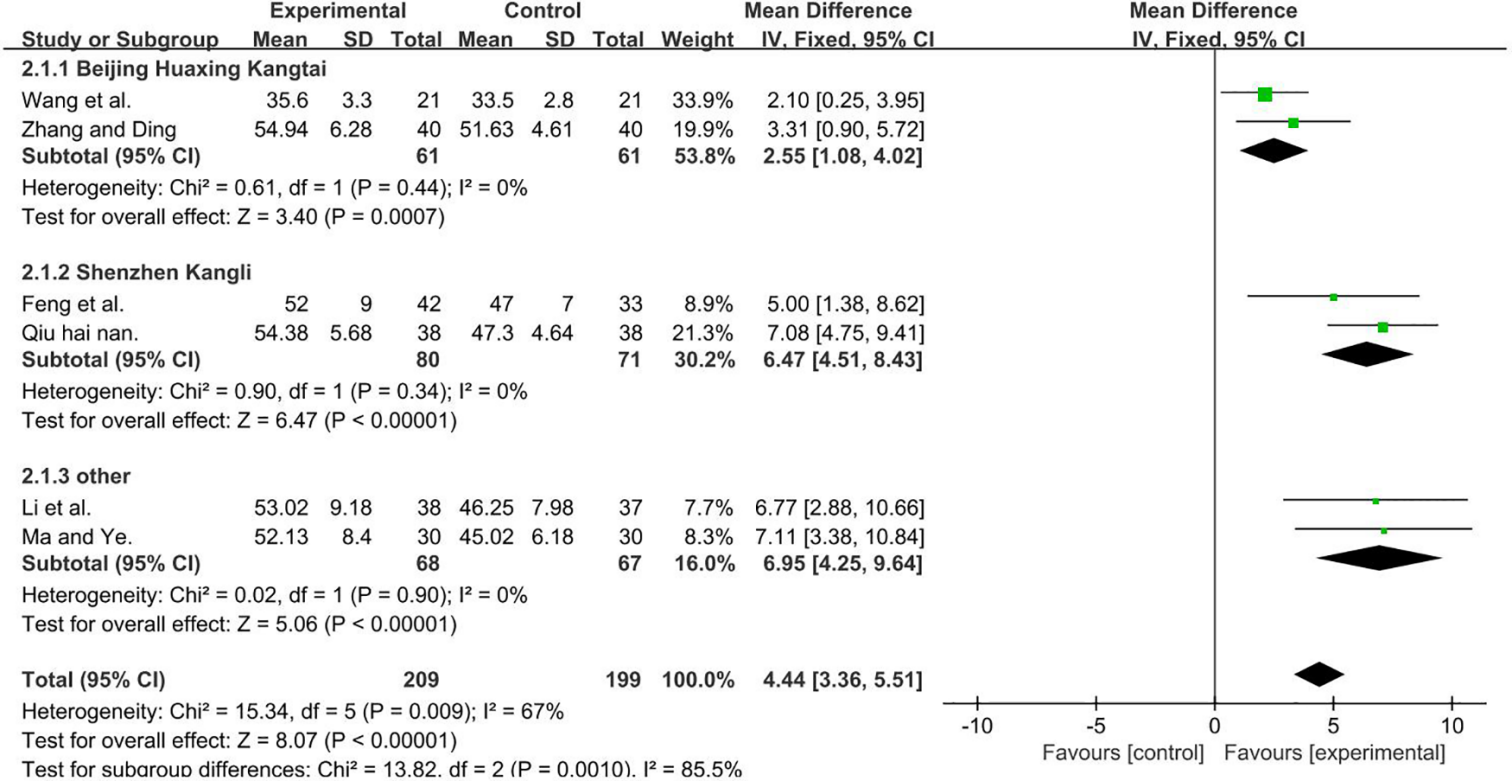
Forest plot showing MD (with 95% CI) for GMFM-B of the included studies comparing the experimental and control groups. Note: For the Beijing Huaxing Kangtai subgroup, *P* = 0.44 and *I*^2^_ _= 0%., fixed-effect model [MD = 2.55, 95% CI (1.08, 4.02); *P* < 0.001]. For the Shenzhen Kangli subgroup, *P* = 0.34 and *I*^2^ = 0%, fixed-effect model [MD = 6.47; 95% CI (4.51, 8.43); *P* < 0.001]. For the other subgroup, *P* = 0.9, and *I*^2^_ _= 0%, fixed-effect model [MD = 6.95; 95% CI (4.25, 9.64); *P* < 0.001]. The analysis results of the three subgroups were statistically significant.

A total of 408 participants were included in six studies (35, 37, 50, 51, 53, 56) [*I*2 = 67%; MD = 4.44; 95% CI (3.36, 5.51); *P* < 0.001]. Heterogeneity was found, and thus, we performed a subgroup analysis. According to the manufacturer of TMS, the group was divided into three subgroups: Beijing Huaxing Kangtai (35, 51), Shenzhen Kangli (53, 56), and others (37, 50), the result favors rTMS, as shown in Figure 5.

##### 3.5.1.3. C

**Figure 6:**
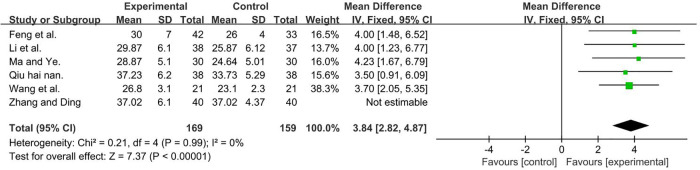
Forest plot showing MD (with 95% CI) for GMFM-C of the included studies comparing the experimental and control groups. Note: (*P* = 1 and *I*^2 ^= 0%), fixed-effect model [MD = 3.84, 95% CI (2.82, 4.87); *P* < 0.001]. The analysis results of three subgroups were statistically significant.

A total of 408 participants were included in six studies (35, 37, 50, 51, 53, 56) [*I*2 = 75%; MD = 4.88, 95% CI (3.89, 5.87); *P* < 0.001]. Heterogeneity was found. Further sensitivity analysis revealed that one study (51) (Zhang Yu Qiong and Ding Jian Ying) used six frequencies, which are recycled (5, 10, 20, 30, 40, and 50 Hz) and differed from other studies that only had one frequency. This was analyzed as a possible cause of heterogeneity, and the analysis was performed after its removal, and the result favors rTMS, as shown in Figure 6.

##### 3.5.1.4. D

**Figure 7:**
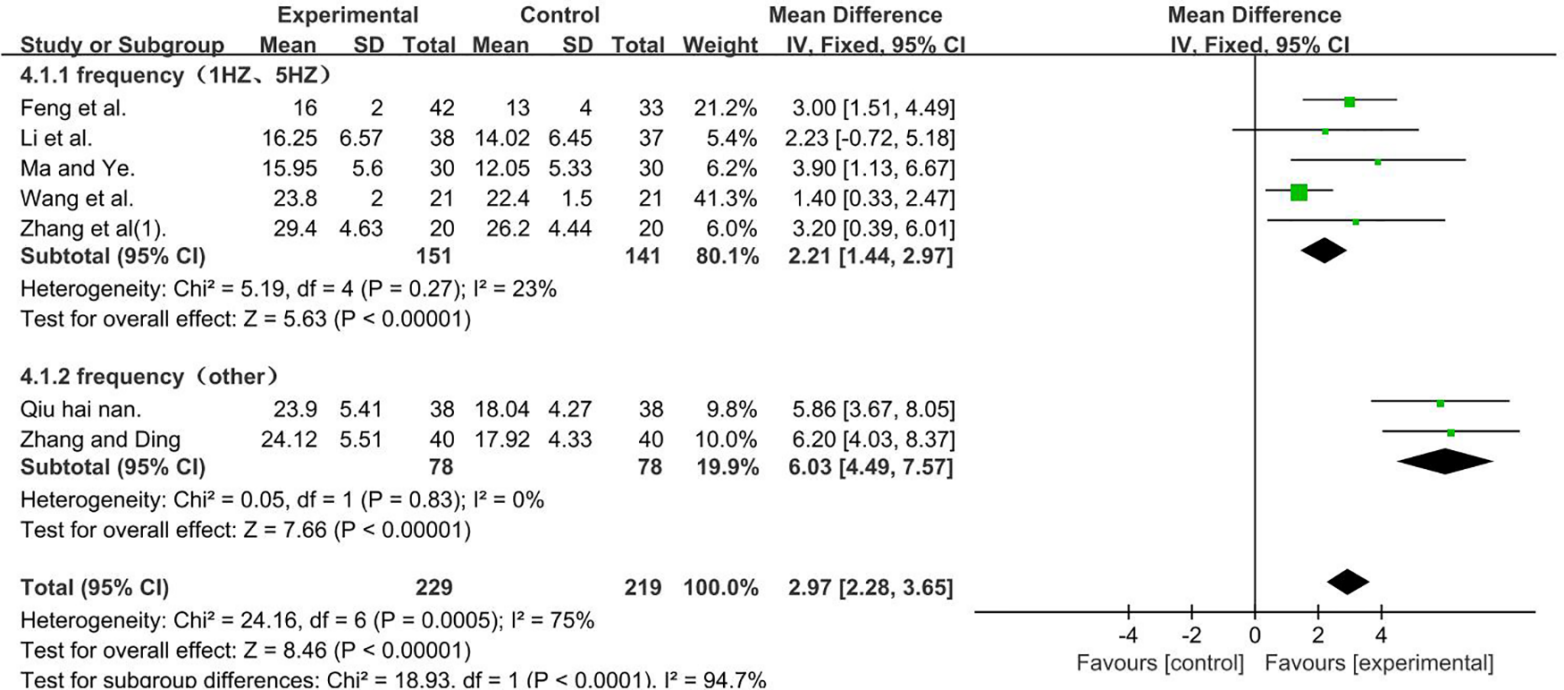
Forest plot showing MD (with 95% CI) for GMFM-D of the included studies comparing the experimental and control groups. Note: For the subgroup with frequencies of 1 and 5 HZ, *P* = 0.27 and *I*^2^ = 23%. The fixed-effect model [MD = 2.21, 95% CI (1.44,2.97); *P* < 0.001]. For the subgroup with the frequency of other, *P* = 0.83 and *I*^2 ^= 0%. fixed-effect model [MD = 6.03, 95% CI (4.49, 7.57); *P* < 0.001]. The analysis results of the two subgroups were statistically significant.

A total of 448 participants were included in seven studies (35, 37, 41, 50, 51, 53, 56); *I*2^ ^= 75% [MD = 2.97, 95% CI (2.28, 3.65); *P* < 0.001]. Heterogeneity existed. We performed a subgroup analysis. According to the frequency of TMS, the group was divided into two subgroups: 1 and 5 HZ (35, 37, 41, 50, 56) and other (51, 53), and the result favors rTMS, as shown in Figure 7.

##### 3.5.1.5. E

**Figure 8:**
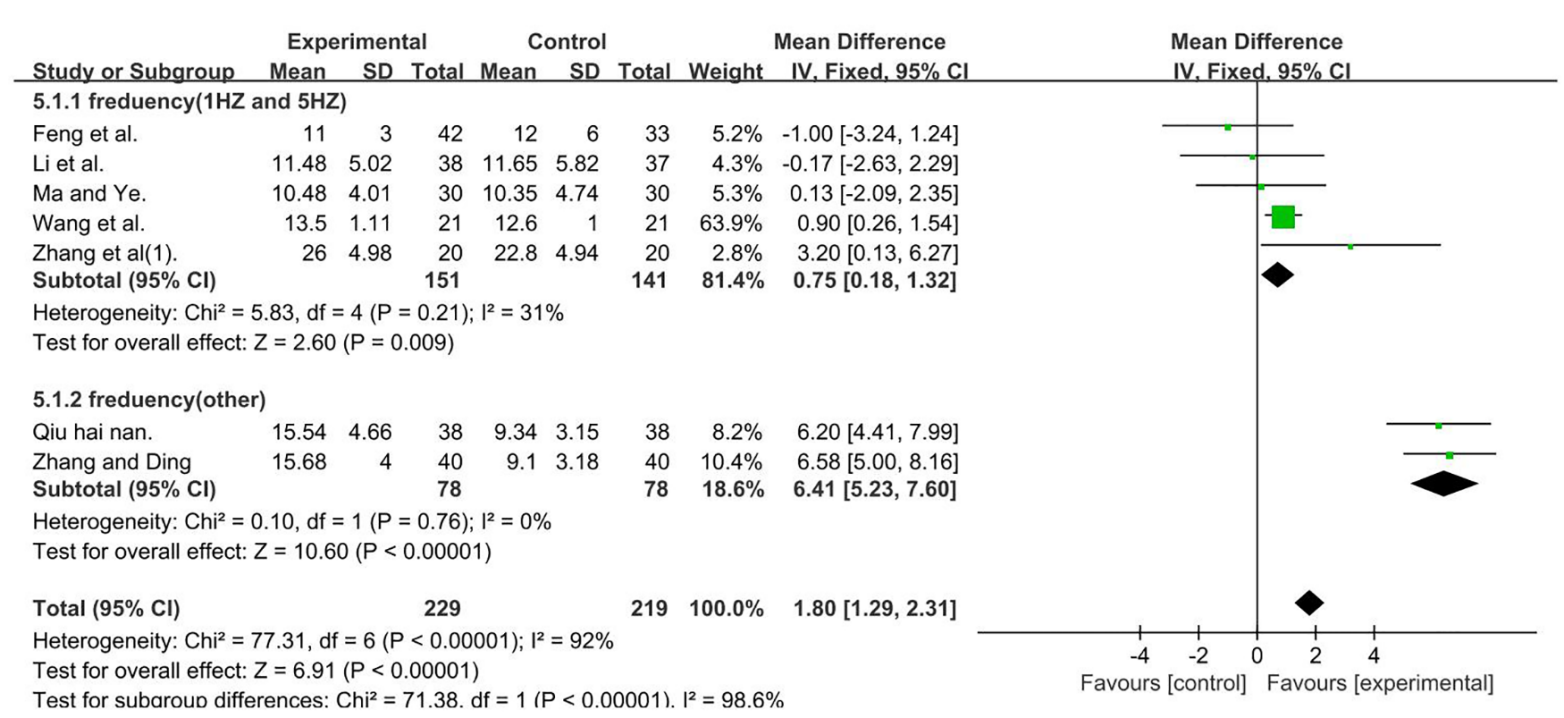
Forest plot showing MD (with 95% CI) for GMFM-E of the included studies comparing the experimental and control groups. Note: For the subgroup with frequencies of 1 and 5 Hz, *P* = 0.21 and *I*^2^ = 31%. fixed-effect model [MD = 0.75, 95% CI (0.18, 1.32); *P* < 0.05]. For the subgroup with frequency (other), *P* = 0.76 and *I*^2^ = 0%, fixed-effect model [MD = 6.41; 95% CI (5.23, 7.60); *P* < 0.001]. The analysis results of the two subgroups were statistically significant.

A total of 448 participants were included in seven studies (35, 37, 41, 50, 51, 53, 56); *I*2^ ^= 92% [MD = 1.80, 95% CI (1.29, 2.31); *P* < 0.001]. Heterogeneity existed. We performed a subgroup analysis. According to the frequency of TMS, the group was divided into two subgroups, namely, 1 and 5 HZ (35, 37, 41, 50, 56) and other (51, 53), and the result favors rTMS, as shown in Figure 8.

##### 3.5.1.6. ALL

**Figure 9:**
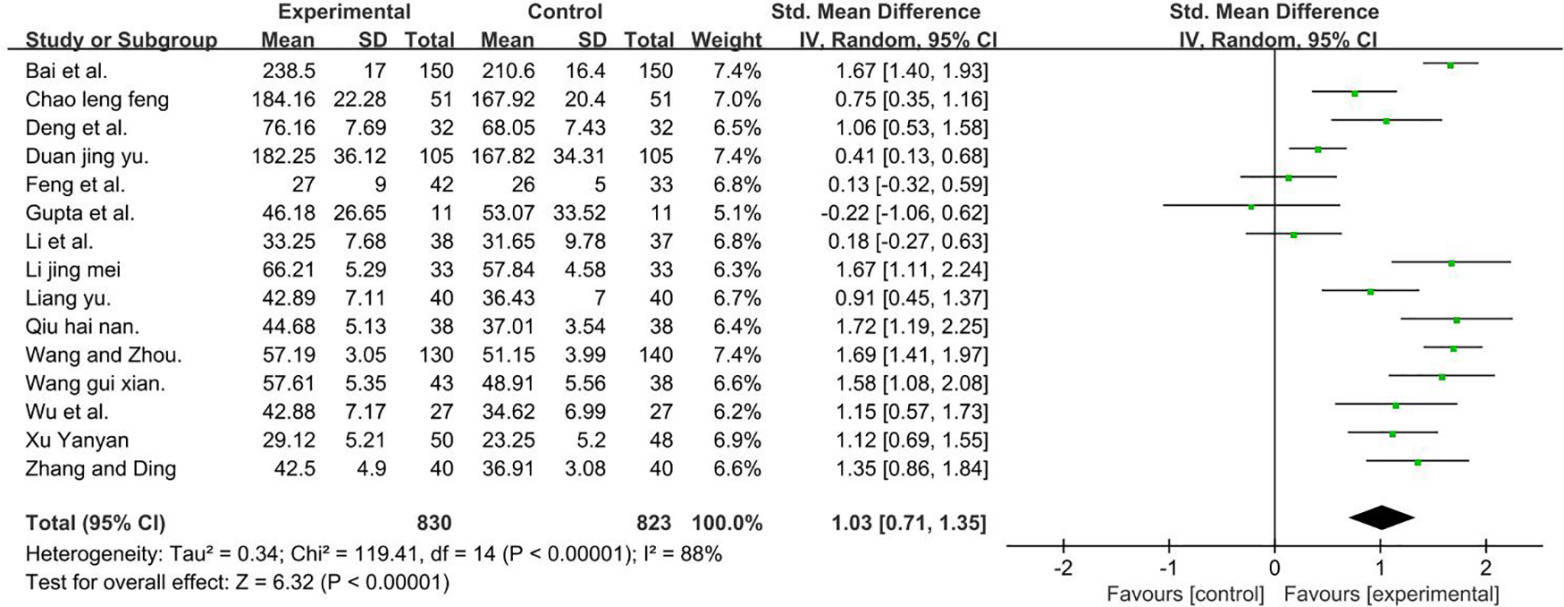
Forest plot showing MD (with 95% CI) for GMFM-ALL of the included studies comparing the experimental and control groups. Note: [*I*^2^ = 88%; MD = 1.03; 95% CI (0.71, 1.35); *P* < 0.001]. The analysis results were statistically significant.

A total of 1,653 participants were included in 11 studies (27, 37, 38, 40, 43–45, 47–49, 51, 53–56) [*I*2 = 88%; MD = 1.09, 95% CI (0.99, 1.20); *P* < 0.001]. Heterogeneity was found. Through subgroup and sensitivity analyses, no significant change in heterogeneity was found. We selected the random-effect model, and the result favors rTMS, as shown in Figure 9.

#### 3.5.2. GDDS

The GDDS had five domains, namely, adaptability, gross motor, fine motor, language, and personal–social responses. Analysis was conducted according to the five domains and the total score.

**Figure 10:**
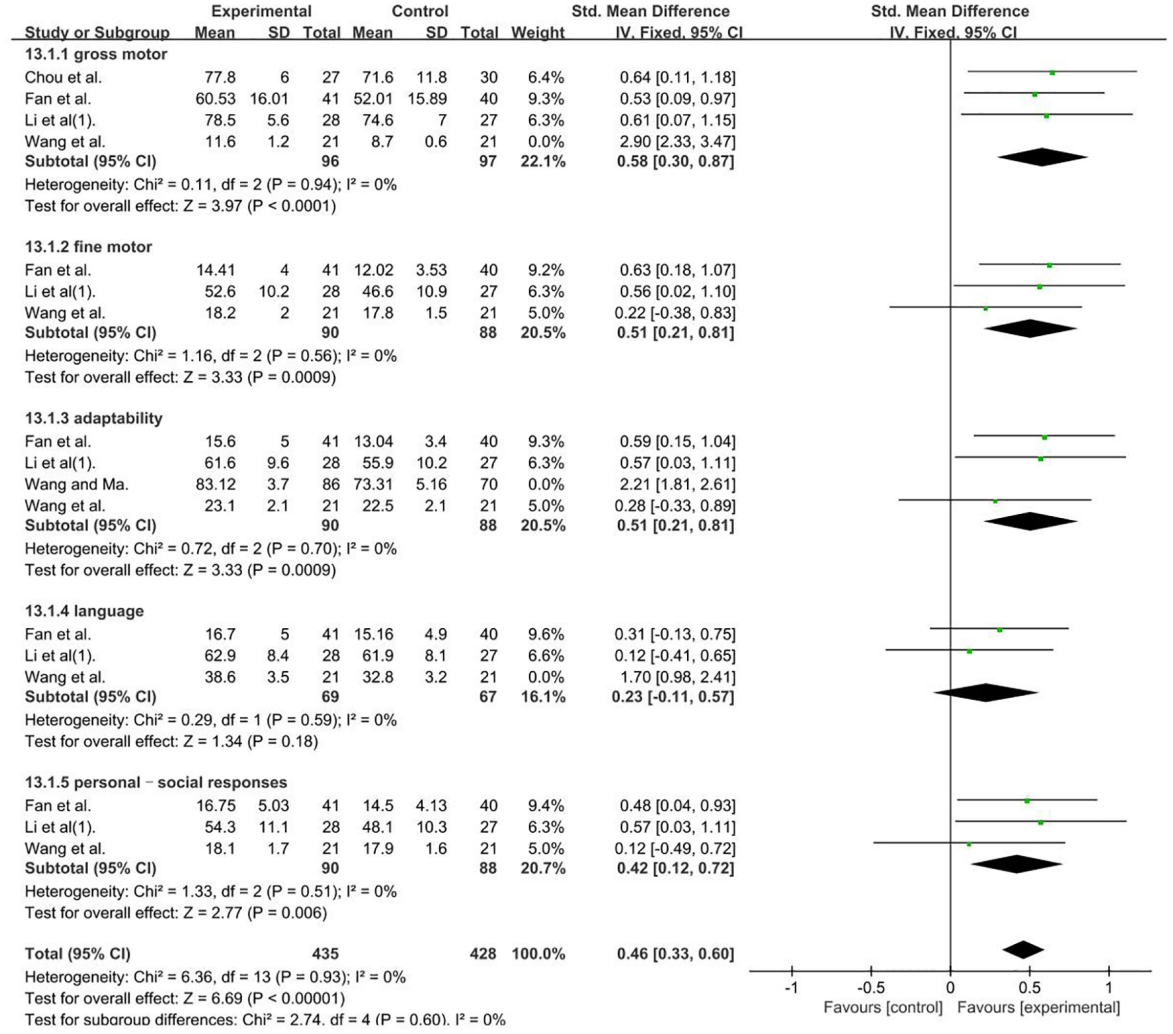
Forest plot showing SMD (with 95% CI) for GDDS of the included studies comparing the experimental and control groups. Note: Gross motor: fixed-effect model [MD = 0.58 95% CI (0.30, 0.87); *P* < 0.001]; GDDS—fine motor: fixed-effect model [MD = 0.51; 95% CI (0.21, 0.81); *P* < 0.05]. GDDS–adaptability: fixed-effect model [SMDs = 0.51; 95% CI (0.21, 0.81); *P* < 0.001]. GDDS–language: fixed-effect model [SMDs = 0.23, 95% CI (0.11, 0.57); *P* = 0.18]. GDDS–personal–social responses: fixed-effect model [SMDs = 0.42; 95% CI (0.12, 0.72); *P* < 0.05]. The analysis results were statistically significant.

GDDS—gross motor, a total of 235 participants were included in five studies (35, 46, 57, 60). Data were compared using different scales; thus, we calculated pooled statistics with SMDs [*I*2 = 88%; SMDs = 1.11; 95% CI (0.29, 1.94); *P* < 0.001]. Heterogeneity was found. Further sensitivity analysis revealed that one study (35) (Wang Li Fan et al.) used the DA of GDDS and differed from other studies, which used the DQ of GDDS, and was considered a possible cause of heterogeneity. The analysis was performed after its removal, as well as selected means, and standard deviations (MD) (*P* = 0.94 and *I*2 = 0%). GDDS—fine motor, a total of 178 participants were included in three studies (35, 46, 57) (*P* = 0.56 and *I*2 = 0%). GDDS–adaptability, a total of 334 participants were included in four studies (35, 46, 57, 59). Given that data were compared using different scales, we calculated pooled statistics according to SMDs [*I^2^* = 93%; SMDs = 1.12; 95% CI (0.88, 1.36); *P* < 0.001]. Heterogeneity was found. Further sensitivity analysis revealed that one study (59) (Wang Ying Hong and Ma Bing Xiang) had an extremely large sample size, which was considerably higher than the those of the other studies. This factor was analyzed as a possible cause of heterogeneity, and analysis was performed after its removal. SMDs were selected (*P* = 0.70 and *I*2 = 0%). GDDS–language, a total of 178 participants were included in three studies (35, 46, 57). Given that data were compared using different scales, we calculated pooled statistics by using SMDs [*I*2 = 85%, SMDs = 0.50; 95% CI (0.19, 0.80); *P* < 0.001]. Heterogeneity was found. Further sensitivity analysis revealed that one study (35) (Wang Li Fan et al.) used the cerebral hemisphere motor cortex site of stimulation in contrast to the other studies, which used the bilateral cerebral motor cortex. This factor was analyzed as a possible cause of heterogeneity, and analysis was performed after its removal. SMDs were selected (*P* = 0.59 and *I*2 = 0%). GDDS–personal–social responses, a total of 178 participants were included in three studies (35, 46, 57). Given that data were compared using different scales, we calculated pooled statistics by using SMDs (*P* = 0.51 and *I*2 = 0%). The analysis results were statistically significant and those results favors rTMS, as shown in Figure 10.

#### 3.5.3. FMFM

**Figure 11:**
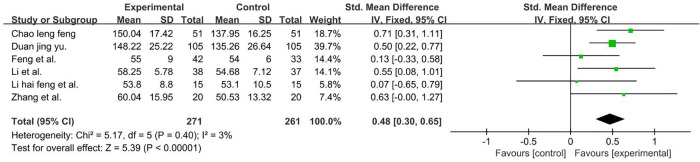
Forest plot showing MD (with 95% CI) for FMFM of the included studies comparing the experimental and control groups. Note: fixed-effect model [SMDs = 0.48; 95% CI (0.30, 0.65); *P* < 0.001]. The analysis results were statistically significant.

A total of 532 participants were included in six studies (37, 39, 42, 54–56). Given that data were compared using different scales, we calculated pooled statistics by using SMDs (*P* = 0.40 and *I*2 = 3%). We used a fixed-effect model and the result favors rTMS, as shown in Figure 11.

#### 3.5.4. PDMS

The PDMS included four domains of the study, namely, grasping, visual–motor integration, Gross Motor Quotient (GMQ), and Fine Motor Quotient (FMQ) score (63). The analysis was performed according to the four domains.

**Figure 12:**
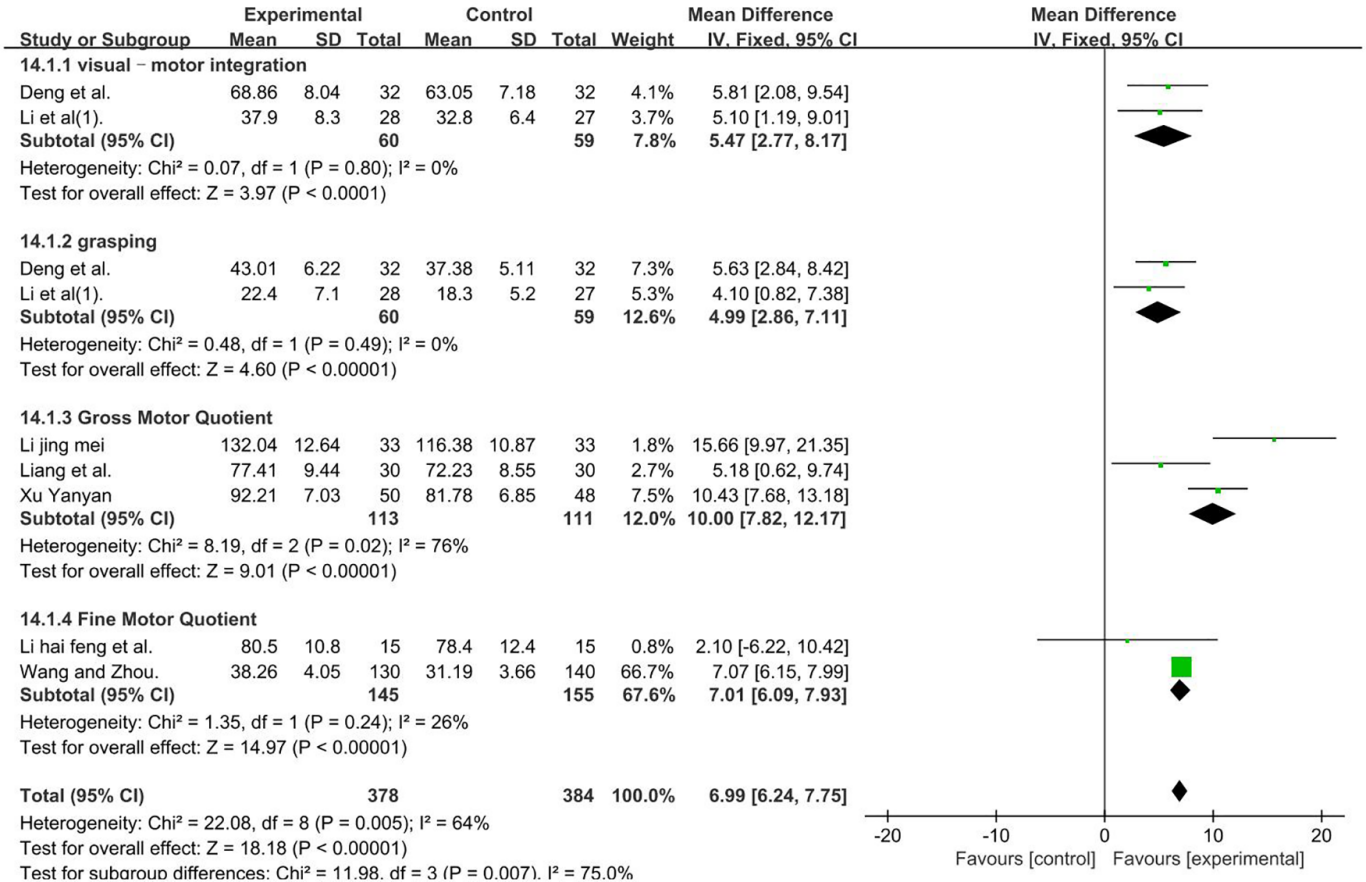
Forest plot showing MD (with 95% CI) for PDMS of the included studies comparing the experimental and control groups. Note: PDMS–visual–motor integration: fixed-effect model [MD = 5.47; 95% CI (2.77, 8.17); *P* < 0.01]. PDMS–grasping: fixed-effect model [MD = 4.99; 95% CI (2.86, 7.11); *P* < 0.01]. PDMS–FMQ: random-effect model [*I*^2^ = 76%; MD = 10.20; 95% CI (5.24, 15.15); *P* < 0.01]. PDMS–GMQ: fixed-effect model [MD = 7.01; 95% CI (6.09, 7.93); *P* < 0.01]. Those analysis results were statistically significant.

PDMS–visual–motor integration, a total of 119 participants were included in two studies (46, 47) (*P* = 0.80 and *I*2 = 0%). We used a fixed-effect model. PDMS–grasping, a total of 119 participants were included in two studies (46, 47) (*P* = 0.49 and *I*2 = 0%). We used a fixed-effect model. PDMS–FMQ, a total of 224 participants were included in three studies (36, 44, 48) [*I*2 = 76%; MD = 10.00; 95% CI (7.82, 12.17); *P* < 0.01]. Subgroup and sensitivity analyses found no significant change in heterogeneity. We selected the random-effect model. PDMS–GMQ, a total of 300 participants were included in two studies (38, 42) (*P* = 0.24 and *I*2 = 26%). We used a fixed-effect model. Those result favors rTMS, as shown in Figure 12.

#### 3.5.5. MAS

**Figure 13:**
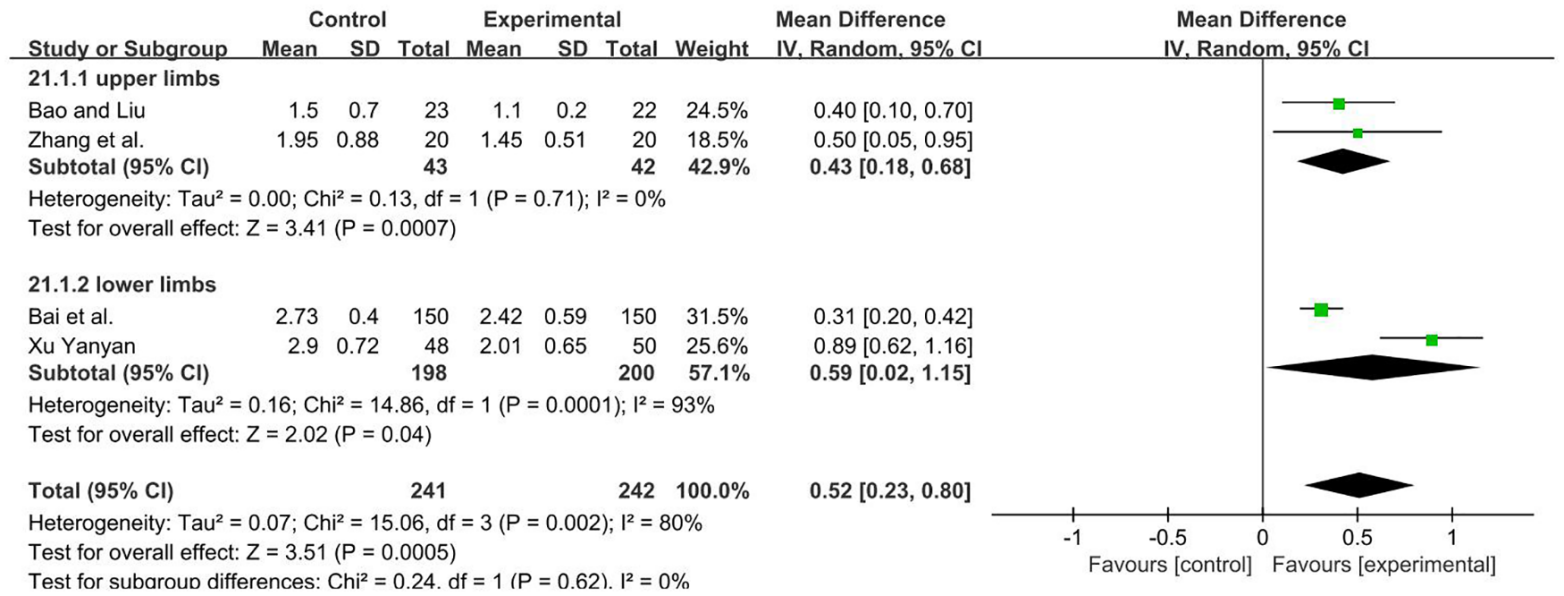
Forest plot showing MD (with 95% CI) for MAS of the included studies comparing the experimental and control groups. Note: For the upper limb subgroup, fixed-effect model [MD = 0.43; 95% CI (0.18, 0.68); *P* < 0.001]. For the lower limbs subgroup, random-effect model [MD = 0.59; 95% CI (0.02, 1.15); *P* < 0.05]. The analysis results of two subgroups were statistically significant.

A total of 483 participants were included in four studies (39, 44, 49, 52) [*I*2 = 80%; MD = 0.40; 95% CI (0.31, 0.50); *P* < 0.001]. Heterogeneity existed. We performed a subgroup analysis. According to the site of muscle spasm test, the group was divided into two subgroups, namely, the upper and lower limbs. For the upper limb subgroup (39, 52), *P* = 0.71 and *I*^2^ = 0%. We selected the fixed-effect model. For the lower limbs subgroup (44, 49), *I*2 = 93%. The high heterogeneity found in the analysis may be due to the high sample size of the study (49), we used a random-effect model. The analysis results of two subgroups were statistically significant and those results favors rTMS, as shown in Figure 13.

#### 3.5.6. S-S

This research analyzed the language situation according to three aspects, namely, language improvement rate, expression quotient, and comprehension quotient.

##### 3.5.6.1. Language improvement rate

**Figure 14:**
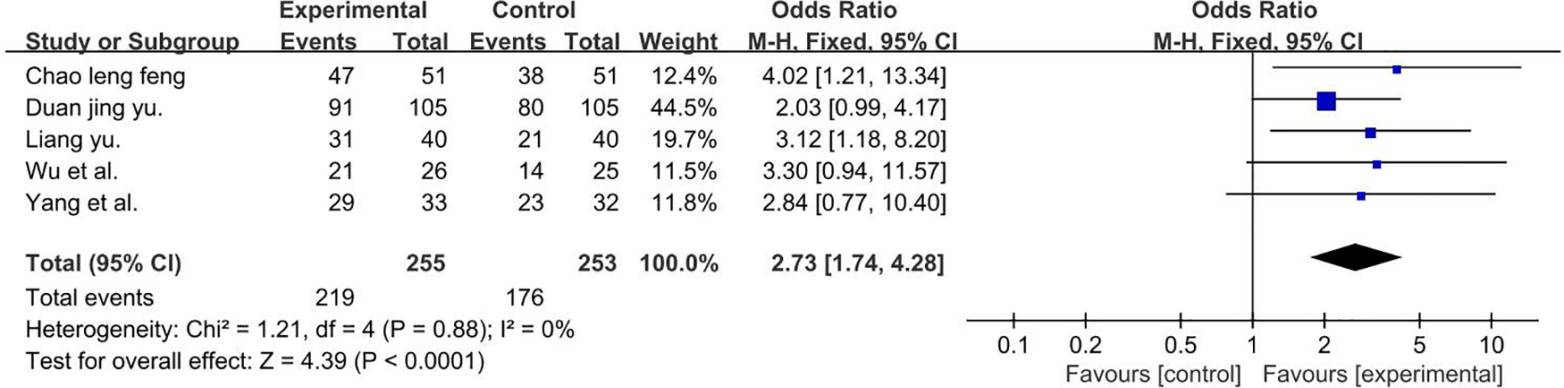
Forest plot showing MD (with 95% CI) for language improvement rate of the included studies comparing the experimental and control groups. Note: fixed-effect model [MD = 2.73; 95% CI (1.74, 4.28); *P* < 0.01]. The analysis results were statistically significant.

A total of 508 participants were included in five studies (40, 43, 54, 55, 62) (*P* = 0.88 and *I*2 = 0%). We used a fixed-effect model and the result favors rTMS, as shown in Figure 14.

##### 3.5.6.2. Comprehension quotient and expression quotient

**Figure 15:**
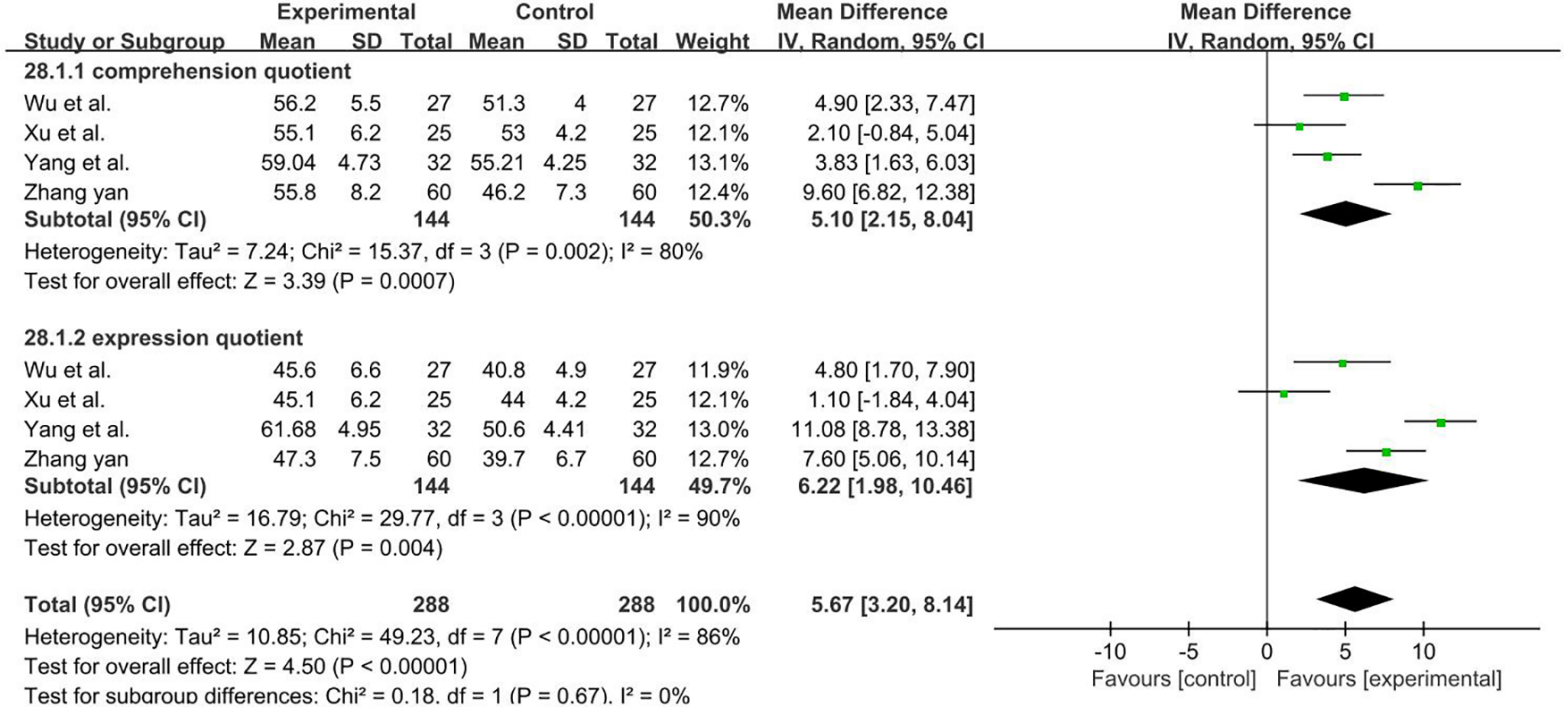
Forest plot showing MD (with 95% CI) for comprehension quotient and expression quotient of the included studies comparing the experimental and control groups. Note: Random-effect model, the result of Comprehension quotient [*I*^2^ = 80%; MD = 5.10; 95% CI (2.15, 8.04); *P* < 0.01], the result of Expression quotient [*I*^2^ = 90%; MD = 6.22; 95% CI (1.98, 10.46); *P* < 0.01]. The analysis results were statistically significant.

A total of 288 participants were included in four studies (40, 58, 61, 62). Subgroup and sensitivity analyses found no significant change in heterogeneity. We selected the random-effect model, and the result favors rTMS, as shown in Figure 15.

#### 3.5.7. Funnel chart

Among all the outcome indicators, only FMFM-ALL included more than 10 studies (15 included studies). Thus, the funnel chart analysis was performed on this outcome indicator. Seven studies were outside the 95% interval, and the two sides of the funnel chart were asymmetrical. These results all showed heterogeneity, as shown in Supplementary Figure S1.

#### 3.5.8. Grade

The GRADEpro GDT online tool was used to evaluate the quality of evidence for the included study outcome indicators. A total of 31 outcome indicators were included, namely, 22 low quality indicators, seven moderate quality indicators, and two very low quality indicators, as shown in Table 4.

**TABLE 4:** The quality of evidence for the included study outcome indicators.

Certainty assessment							№ of patients		Effect		Certainty	Importance
№ of studies	Study design	Risk of bias	Inconsistency	Indirectness	Imprecision	Other considerations	rTMS	control	Relative (95% CI)	Absolute (95% CI)		
**GMFM-A**												
6	randomised trials	very serious^a^	serious^b^	not serious	not serious	none	199	209	–	MD **1.24 lower** (1.85 lower to 0.62 lower)	⊕◯◯◯ VERY LOW	
**GMFM-A - 30min**												
3	randomised trials	serious^a^	not serious	not serious	not serious	none	111	120	–	MD **0.86 lower** 1.52 lower to 0.21 lower)	⊕⊕⊕◯ MODERATE	
**GMFM-A - 20min**												
3	randomised trials	serious^a^	not serious	not serious	serious^c^	none	88	89	–	MD **4.32 lower** 6.2 lower to 2.43 lower)	⊕⊕◯◯ LOW	
**GMFM-B**												
6	randomised trials	serious^a^	serious^b^	not serious	not serious	none	199	209	–	MD **4.44 lower** (5.51 lower to 3.36 lower)	⊕⊕◯◯ LOW	
**GMFM-B - Beijing Huaxing Kangtai**												
2	randomised trials	serious^a^	not serious	not serious	serious^c^	none	61	61	–	MD **2.55 lower** (4.02 lower to 1.08 lower)	⊕⊕◯◯ LOW	
**GMFM-B - Shenzhen Kangli**												
2	randomised trials	serious^a^	not serious	not serious	serious^c^	none	71	80	–	MD **6.47 lower** (8.43 lower to 4.51 lower)	⊕⊕◯◯ LOW	
**GMFM-B – other**												
2	randomised trials	serious^a^	not serious	not serious	serious^c^	none	67	68	–	MD **6.95 lower** (9.64 lower to 4.25 lower)	⊕⊕◯◯ LOW	
**GMFM-C**												
5	randomised trials	serious^a^	not serious	not serious	not serious	none	159	169	–	MD **3.82 lower** (4.92 lower to 2.73 lower)	⊕⊕⊕◯ MODERATE	
**GMFM-D**												
7	randomised trials	serious^a^	serious^b^	not serious	not serious	none	219	229	–	MD **2.97 lower** (3.65 lower to 2.28 lower)	⊕⊕◯◯ LOW	
**GMFM-D - frequency (1HZ, 5HZ)**												
5	randomised trials	serious^a^	not serious	not serious	not serious	none	141	151	–	MD **2.21 lower** (2.97 lower to 1.44 lower)	⊕⊕⊕◯ MODERATE	
**GMFM-D - frequency (other)**												
2	randomised trials	serious^a^	not serious	not serious	serious^c^	none	78	78	–	MD **6.03 lower** (7.57 lower to 4.49 lower)	⊕⊕◯◯ LOW	
**GMFM-E**												
7	randomised trials	serious^a^	serious^b^	not serious	not serious	none	219	229	–	MD **1.8 lower** (2.31 lower to 1.29 lower)	⊕⊕◯◯ LOW	
**GMFM-E - freduency (1HZ and 5HZ)**												
5	randomised trials	serious^a^	not serious	not serious	not serious	none	141	151	–	MD **0.75 lower** (1.32 lower to 0.18 lower)	⊕⊕⊕◯ MODERATE	
**GMFM-E - freduency (other)**												
2	randomised trials	serious^a^	not serious	not serious	serious^c^	none	78	78	–	MD **6.41 lower** (7.6 lower to 5.23 lower)	⊕⊕◯◯ LOW	
**GMFM-ALL**												
15	randomised trials	serious^a^	serious^b^	not serious	not serious	publication bias strongly suspected ^d^	823	830	–	SMD **1.09 lower** (1.2 lower to 0.99 lower)	⊕◯◯◯ VERY LOW	
**GDDS-gross motor**												
3	randomised trials	serious^a^	not serious	not serious	serious^c^	none	97	96	–	MD **5.18 lower** (7.74 lower to 2.62 lower)	⊕⊕◯◯ LOW	
**GDDS-fine motor**												
3	randomised trials	serious^a^	not serious	not serious	serious^c^	none	88	90	–	SMD **0.51 lower** (0.81 lower to 0.21 lower)	⊕⊕◯◯ LOW	
**GDDS-adaptability**												
3	randomised trials	serious^a^	not serious	not serious	serious^c^	none	88	90	–	SMD **0.51 lower** (0.81 lower to 0.21 lower)	⊕⊕◯◯ LOW	
**GDDS-language**												
2	randomised trials	serious^a^	not serious	not serious	serious^c^	none	67	69	–	SMD **0.23 lower** (0.57 lower to 0.11 higher)	⊕⊕◯◯ LOW	
**GDDS-personal-social response**												
3	randomised trials	serious^a^	not serious	not serious	serious^c^	none	88	90	–	SMD **0.42 lower** (0.72 lower to 0.12 lower)	⊕⊕◯◯ LOW	
**FMFM**												
6	randomised trials	serious^a^	not serious	not serious	not serious	none	261	271	–	SMD **0.48 lower** (0.65 lower to 0.3 lower)	⊕⊕⊕◯ MODERATE	
**PDMS-visual-motor integration**												
2	randomised trials	serious^a^	not serious	not serious	serious^c^	none	59	60	–	MD **5.47 lower** (8.17 lower to 2.77 lower)	⊕⊕◯◯ LOW	
**PDMS-grasping**												
2	randomised trials	serious^a^	not serious	not serious	serious^c^	none	59	60	–	MD **4.99 lower** (7.11 lower to 2.86 lower)	⊕⊕◯◯ LOW	
**PDMS-FMQ**												
3	randomised trials	serious^a^	serious^b^	not serious	not serious	none	111	113	–	MD **10.2 lower** (15.15 lower to 5.24 lower)	⊕⊕◯◯ LOW	
**PDMS-GMQ**												
2	randomised trials	serious^a^	not serious	not serious	not serious	none	155	145	–	MD **7.01 lower** (7.93 lower to 6.09 lower)	⊕⊕⊕◯ MODERATE	
**MAS**												
4	randomised trials	serious^a^	serious^b^	not serious	not serious	none	241	242	–	MD **0.52 higher** (0.23 higher to 0.8 higher)	⊕⊕◯◯ LOW	
**MAS - upper limbs**												
2	randomised trials	serious^a^	not serious	not serious	serious^c^	none	43	42	–	MD **0.43 higher** (0.18 higher to 0.68 higher)	⊕⊕◯◯ LOW	
**MAS - lower limbs**												
2	randomised trials	serious^a^	serious^b^	not serious	not serious	none	198	200	–	MD **0.59 higher** (0.02 higher to 1.15 higher)	⊕⊕◯◯ LOW	
**S-S language improvement rate**												
5	randomised trials	serious^a^	not serious	not serious	not serious	none	176/253 (69.6%)	219/255 (85.9%)	**OR 0.37** (0.23 to 0.57)	166 fewer per 1,000 (from 276 fewer to 83 fewer)	⊕⊕⊕◯ MODERATE	
**S-S comprehension quotient**												
4	randomised trials	serious^a^	serious^b^	not serious	not serious	none	144	144	–	MD **5.01 lower** (6.29 lower to 3.72 lower)	⊕⊕◯◯ LOW	
**S-S expression quotient**												
4	randomised trials	serious^a^	serious^b^	not serious	not serious	none	144	144	–	MD **6.22 lower** (10.46 lower to 1.98 lower)	⊕⊕◯◯ LOW	

^a^The included studies (suspected) are biased in randomization, blinding, allocation concealment, or selective reporting. ^b^The confidence interval overlap is poor, the I2 value of the combined result is large, and the heterogeneity is moderate. ^c^The confidence interval is not narrow enough or there are few included studies. ^d^The funnel graph is asymmetric, and there is a possibility of publication offset.

## 4. Discussion

This meta-analysis of the study included 29 studies. According to the PEDro scale, only four studies had excellent quality (26, 32–34), whereas 10 studies had low quality (27, 35, 41, 43, 44, 48, 50, 51, 55, 56). According to the results of evaluation using the Cochrane Collaborative Network Bias Risk Assessment Scale, 10 studies (27, 35, 41, 43–45, 50, 51, 55, 56) did not report random sequence generation, two studies (34, 54) described allocation concealment, four studies (26, 32–34) described the procedures for blinding participants and persons, and six studies (26, 32, 36, 38, 45, 59) explained that the assessment of outcome measures was blinded. The abovementioned issues affected the quality of the results and the risk of bias. The results of GRADE's quality of evidence showed that the main outcome indicators had low quality, and two outcome indicators had very low quality. Seven outcome indicators had moderate quality, and no high quality outcome indicator was found. Overall, the quality of the outcome indicators was low, and the reasons were as follows. 1. Allocation concealment and blinding in the experimental design in the included literature was not strictly controllable. 2. The heterogeneity was biased, which may be related to the prescription of intervention factors, such as differences in stimulation frequency and time. 3. The size of the included literature and the sample size were small.

In the study characteristics, most studies focused on the comparison between conventional rehabilitation and conventional rehabilitation combined with rTMS. Only four studies (26, 32–34) described the comparison between sham and real TMS, and the above two research methods showed the effectiveness of rTMS in improving motor function and language ability in patients with CP. The included studies examined the site of stimulation, the duration of rTMS, and the number of rTMS sessions; stimulation frequencies of TMS were 0.2 (53), 1 (33), 5 (27), 10 (50), and 30 Hz (60). Studies on the efficacy of rTMS at different frequencies are few, and no clear evidence that stimulation of frequency contributes to CP treatment is available. High-frequency rTMS (stimulation rate >1 Hz), produces an excitatory after effect (64, 65). Conversely, low-frequency rTMS (stimulation rate ≤ 1 Hz) depresses excitability (26, 66, 67). This is applicable to stroke patients, and whether it is applicable to patients with CP remains to be discovered. Valle et al. (32) reported the stimulation frequency of rTMS (sham vs. 1 Hz vs. 5 Hz) and showed significant reduction in spasticity after 5 Hz. Gupta et al. (1) reported repetitive transcranial magnetic stimulation pulses (1,500 vs. 2,000 vs. 2500) and showed that the overall improvement rates in motor functions were 2.33% in 1,500 pulses, 3.58% in 2000 pulses, and 5.17% in 2,500 pulses. The 2,500 pulse groups showed significant improvement in motor function. Therefore, the efficacy of rTMS in the treatment of CP is affected by factors, such as stimulation frequency, intensity, duration, and pulse sequence.

Motor dysfunction is among the common symptoms of CP. Therefore, many studies on motor dysfunction in patients with CP have been conducted. In our review, GMFM, FMFM, PDMS, and GDDS were used in evaluating results. GMFM is a criterion-referenced observational measure for assessing gross motor function in children with CP. It is a reliable method for assessing the gross motor functional ability and quality of movement in children with CP (68). Our review of the five domains of GMFM, namely, (A) lying and rolling, (B) sitting, (C) crawling/kneeling, (D) standing, and (E) walking/running/jumping, showed that rTMS can improve the aspects of gross motor function. In addition, the result of GDDS-gross motor showed that rTMS can improve these aspects in children with CP. For the evaluation of the fine motor of patients with CP, we used FMFM and PDMS. TMS can improve the aspects of fine motor function. The result of GDDS-fine motor showed that rTMS can improve the aspects of fine motor function in children with CP. Marzbani et al. (14) demonstrated the 1 Hz rTMS could improve motor function in children with CP. Dadashi et al. (67) showed that after 3 weeks of rTMS training, the balance control of children with CP can improve, indicating that rTMS may improve balance control by promoting the function of corticospinal tract and ascending pathways. However, studies with larger sample sizes are needed to confirm this finding.

Spasticity is the main cause of motor function disability in children with CP (69). It is an important factor affecting the quality of life of patients with CP, because long-term spasticity can lead to musculoskeletal complications, such as contracture, pain, and subluxation. In addition, the elimination of spasms can improve the motor function of these patients (70). Our review included four studies (39, 44, 49, 52) [*I*2 = 80%; MD = 0.40; 95% CI (0.31, 0.50); *P* < 0.0001], and the analysis results for these studies were statistically significant. Guptal et al. (71) showed that conventional treatment had no obvious effect on the improvement of muscle spasm and that rTMS combined with conventional treatment significantly reduced muscle tightness. In 2019, Guptal et al. (27) compared rTMS and conventional physical therapy. The MAS score of the rTMS treatment group showed that the spasm of the muscles in the lower extremity was significantly reduced, and the motor function greatly improved. Valle et al. (32) showed that high-frequency stimulation was more effective in improving spasticity, although their evidence was insufficient.

The language development disorder in children with CP may be due to many reasons, such as speech motor control, cognition, language, and sensory/perception (72). European epidemiological data showed that 60% of children with CP have communication disorders (73). In addition, language development disorders can have many adverse effects on children with CP and are not conducive to social communication (74) and quality of life (74). Our review analyzed the language situation from three aspects, namely, language improvement rate, expression quotient, and comprehension quotient. The rTMS can improve the abovementioned various aspects of language ability. Expression and comprehension quotients significantly improved compared with those in the control group. In addition, GDDS results showed that language and personal–social responses were more obviously improved by rTMS. However, studies on the treatment of language disorders with rTMS are few, and the sample size is relatively low. Further expansion and improvement of research are needed.

### 4.1. Study limitations

Our findings are based on articles written in English and Chinese. Articles in other languages were not included, and their exclusion may have implications for our research. In the inclusion of outcome indicators, the data were all derived from the scale. Only S-S was included in the indicators of language ability, which had a certain impact on this study.

## 5. Conclusions

This review suggested that rTMS could improve the motor function and language ability of patients with CP. However, the review indicated large differences among studies in terms of rTMS prescription, particularly in stimulation frequency, intensity, duration, and pulse train. Therefore, the standardization of prescriptions needs to be explored and improved. Studies using large sample size and rigorous research designs are needed to obtain sufficient evidence on the effectiveness of using rTMS to treat patients with CP.

## Data Availability

The original contributions presented in the study are included in the article/Supplementary Material, further inquiries can be directed to the corresponding author/s.
