# Supplementary figures and images for: Effects of repetitive transcranial magnetic stimulation on motor function and language ability in cerebral palsy: A systematic review and meta-analysis
Source: Front Pediatr. 2023 Feb 16;11:835472. doi: 10.3389/fped.2023.835472 (PMC9978792; doi:10.3389/fped.2023.835472)

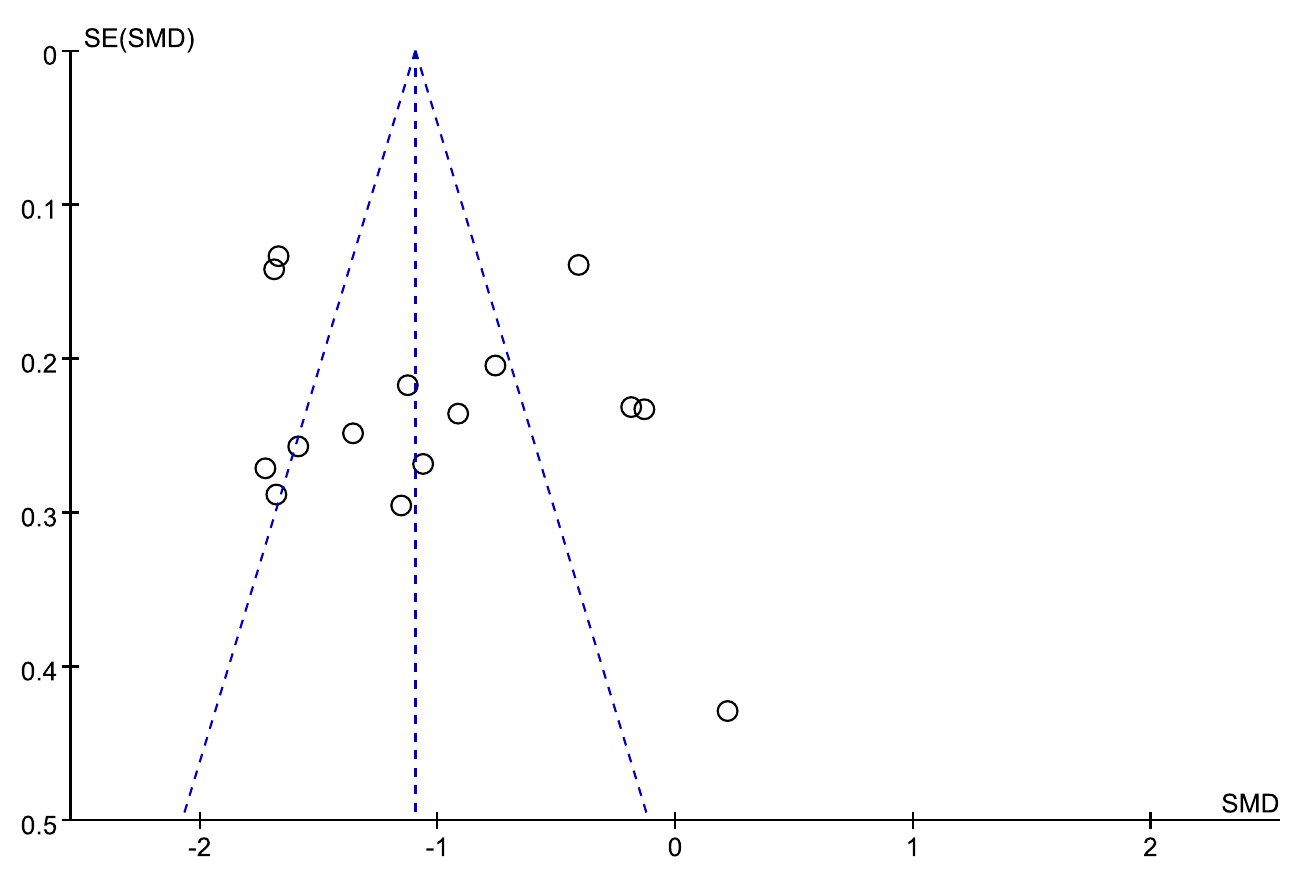

Supplement: Supplementary file 1 [file Image1.jpg]
